# Association between the Interleukin-6 Promoter Polymorphism −174G/C and Serum Lipoprotein(a) Concentrations in Humans

**DOI:** 10.1371/journal.pone.0024719

**Published:** 2011-09-14

**Authors:** Heiner K. Berthold, Matthias Laudes, Wilhelm Krone, Ioanna Gouni-Berthold

**Affiliations:** 1 Research Group on Geriatrics, Evangelical Geriatrics Center Berlin (EGZB), Charité University Medicine Berlin, Berlin, Germany; 2 Lipid Clinic at the Interdisciplinary Metabolism Center, Campus Virchow Clinic, Charité University Medicine Berlin, Berlin, Germany; 3 Center of Endocrinology, Diabetes, and Preventive Medicine, University of Cologne, Cologne, Germany; 4 Department of Internal Medicine I and Cluster of Excellence in Inflammation at Interfaces, University of Kiel, Kiel, Germany; University of Bristol, United Kingdom

## Abstract

**Background:**

Lipoprotein(a) [Lp(a)] is an independent risk factor for cardiovascular disease. The interleukin-6 (IL-6) receptor antagonist tocilizumab has been shown to lower serum Lp(a) concentrations. We investigated whether the IL-6 single nucleotide polymorphism −174G/C is associated with baseline serum Lp(a) concentrations.

**Methodology/Principal Findings:**

We divided 2321 subjects from the Lipid Analytic Cologne (LIANCO) cohort into 2 groups, the ones with substantially elevated Lp(a), defined as concentrations ≥60 mg/dl (*n* = 510), and the ones with Lp(a) <60 mg/dl (*n* = 1811). The association with the genotypes GG (33.7%), GC (50.75%) and CC (15.55%) was investigated. The GC and the CC genotype were associated with a significantly increased odds ratio of having substantially elevated Lp(a) concentrations (OR = 1.3, 95% CI 1.04 to 1.63, P = 0.02 and OR = 1.44, 95% CI 1.06 to 1.93, P = 0.018). These associations remained significant after adjusting for age, sex, smoking behavior, body mass index, serum lipoproteins, hypertension and diabetes. Of these covariates, only LDL cholesterol was significantly and independently associated with elevated Lp(a) concentrations.

**Conclusions/Significance:**

The IL-6 single nucleotide polymorphism −174G/C is associated with increased odds of having elevated Lp(a). Whether this association plays a role in the Lp(a)-lowering effects of IL-6 receptor antagonists remains to be established.

## Introduction

Lipoprotein(a) [Lp(a)] has been considered a cardiovascular risk factor for many years [1]. Together with recent genetic findings and based on robust and specific associations between elevated Lp(a) concentrations and increased cardiovascular disease risk, it is now accepted that Lp(a) is *causally* related to premature cardiovascular disease [2]. A European Atherosclerosis Society Consensus Panel recommends screening for elevated Lp(a) for all patients at intermediate or high cardiovascular risk [2]. Moreover, the panel points to the need for randomized controlled intervention trials with selective reduction in plasma Lp(a) concentrations to reduce cardiovascular disease in both primary and secondary prevention settings, as they are urgently required to define more precisely whom to treat and to what target levels [2].

There is no generally accepted drug treatment for Lp(a) lowering, although some drugs have been described to be effective [3]. Among these are niacin [4], the cholesteryl ester transfer protein (CETP) inhibitor anacetrapib [5], the thyroid hormone analogue eprotirome [6], the apoB synthesis inhibitor mipomersen [7], and hormone replacement therapy or raloxifene, among others [3]. However, no specific therapy primarily targeting Lp(a) is available and no study until now has shown that selectively decreasing Lp(a) will decrease cardiovascular disease risk.

A recent study from our group revealed that the humanized monoclonal antibody against the interleukin-6 (IL-6) receptor, tocilizumab, decreased Lp(a) concentrations in humans [8]. Tocilizumab is approved for the treatment of rheumatoid arthritis and inhibition of IL-6 signaling seems to have multiple metabolic effects, *e.g.* decreasing insulin resistance. Variations caused by IL-6 promoter polymorphisms are associated with diabetes mellitus, obesity and several features of the metabolic syndrome [9]. The IL-6 gene single nucleotide polymorphism (SNP) −174G/C of the 5′ flanking region influences plasma levels of IL-6 [10]–[12]. Based on our discovery that the IL-6 antibody tocilizumab decreases Lp(a), we proceeded to investigate whether the presence of this SNP is associated with baseline Lp(a) concentrations in humans. We were able to confirm our hypothesis that the SNP −174G/C predisposes to elevated Lp(a) levels.

## Methods

### Study design and subjects

The subjects were obtained from the LIANCO database (*Lipid Analytic Cologne*) [13], [14]. In brief, LIANCO was designed to assess the relationship between genetic mutations, serum lipoproteins, other biochemical parameters, and clinical data on atherosclerotic disease, hypertension and diabetes. Approval of the study protocol was obtained from the ethics committee of the medical faculty of the University of Cologne (http://www.medfak.uni-koeln.de/179.html).

Written informed consent was obtained from all subjects. The ethics committee approved the LIANCO study but did not specifically approve the current retrospective study. However, the current study is fully covered by the ethics committee's vote. The study was performed doing a reanalysis of existing LIANCO data, *i.e.* there were no *de novo* experiments on the subjects. Institutional Review Board (IRB) approval for this study was obtained prior to creating the LIANCO registry.

Between spring 1999 and March 2002 a total of about 5000 patients were recruited in the Cologne (Germany) area by hospitals and office-based physicians. Patients' data were recorded, their lipoproteins analyzed and samples frozen for DNA extraction.

Lp(a) was measured using an immunoturbidimetric assay [15] using a COBAS INTEGRA 400 analyser (Roche Diagnostics, Mannheim, Germany). The intra-assay coefficient of variation was 0.7–2.3% and the inter-assay coefficient of variation was 2.9–3.3%.

Lp(a) measurements were available in 3904 subjects and determination of the −174G/C SNP in 2490 subjects. A total of 2321 subjects participated in the current study, the ones in whom both measurements were available. Lp(a) concentrations were similar in the total (*n* = 3904) and in the participating (*n* = 2321) cohort (median [interquartile range] 19.0 [45.0] or 18.0 [42.0] mg/dl , respectively). Genotype frequencies were also similar between the total (*n* = 2490) and the participating (*n* = 2321) cohort (GG, GC and CC 33.49%, 50.88% and 15.62% or 33.49%, 50.87% and 15.64%, respectively).

Atherosclerotic disease was defined as the presence and/or history of ≥1 of the following: history of stroke, transient ischemic attack, prolonged ischemic neurological deficit, coronary heart disease (CHD), and peripheral vascular disease (PAD). CHD was defined as the presence of ≥1 of the following: angiographic evidence of CHD, myocardial infarction, angina pectoris, coronary bypass surgery or positive stress test. Hypertension was defined as known or newly diagnosed hypertension according to current national guidelines, *i.e.* systolic blood pressure values >140 mmHg or diastolic values >90 mmHg. If hypertension was controlled by medication, patients were still considered hypertensive, irrespective of their blood pressure readings.

### Detection of the −174G/C polymorphism

Genomic DNA was prepared from peripheral blood using standard techniques. The −174G/C SNP was detected by PCR followed by restriction fragment length polymorphism as previously described [16]. In short, a 198 bp fragment of the IL-6 gene was amplified. Forward and reverse primer sequences were 5′-TGACTTCAGCTTTACTCTTTGT-3′ and 5′-CTGATTGGAAACCTTATTAGG-3′, respectively. Each PCR cycle consisted of denaturation for 60 seconds at 94°C, annealing for 95 seconds at 55°C, and extension for 60 seconds at 72°C, followed by a final extension at 72°C for nine minutes. PCR products were digested with *Sfa*NI (Fermentas, St. Leon-Rot, Germany) and separated by electrophoresis in 3% agarose. The presence of a single 198 bp band corresponds to the CC genotype; bands at 140 and 58 bp correspond to the GG genotype and the presence of three bands corresponds to the GC genotype.

The PCR products were automatically sequenced (ABI Prism Genetic Analyzer model 310, Applied Biosystems, Foster City, CA). The sequence of both strands was determined.

Quality checks to ensure correctness of the genotypes were carried out by independent rating of the results by two investigators. Discrepancies were resolved by either reaching consensus or re-genotyping.

### Statistical analysis

Statistical analysis was carried out using Stata Version 11 (StataCorp LP, College Station, TX). Statistical significance was defined as P<0.05 and all tests were performed 2-sided. Descriptive statistics are given, unless otherwise indicated, as mean ± SD. Comparison of mean values was performed by unpaired Student's *t*-test. Chi square P values were calculated to test for differences in allele and genotype frequencies and in categorical variables. Allele frequencies were estimated by gene counting.

Two-way analysis of variance was used to establish the influence of categorical variables on continuous parameters. A logistic modeling approach was used to investigate parameters that may be associated with the presence of hypertension, diabetes, or atherosclerotic disease. Multiple logistic regression was used to adjust for the effects of sex, age, body mass index (BMI), and smoking. Lp(a) and triglyceride values were log-transformed before statistical analysis. Original rather than log-transformed values are presented in the results for clarity.

## Results

### Study cohort and biochemical analysis

The characteristics of the 2321 study subjects are presented in **Table 1**. There were 1811 subjects with Lp(a) <60 mg/dl and 510 subjects with Lp(a) ≥60 mg/dl. The level of Lp(a) ≥60 mg/dl was chosen as a cut-off based on the significantly increased risk for CHD above this level, as observed in several recent studies [17]–[19]. There were no differences between the groups in sex distribution, age, smoking behavior, blood pressure or the presence of hypertension. BMI was lower in the group with higher Lp(a). Total cholesterol, LDL and HDL cholesterol concentrations were higher while triglycerides were lower in this group. There were no differences in lipid-lowering medication use. The rate of atherosclerotic disease was significantly higher in the high Lp(a) group (28 *vs.* 18%), while the prevalence of diabetes was lower (13 *vs.* 18%). Of the 2321 study subjects, 687 (29.6%) had Lp(a) concentrations <10 mg/dl, 781 (33.6%) had Lp(a) ≥10 and <30 mg/dl, 343 (14.8%) had Lp(a) ≥30 and <60 mg/dl, 267 (11.4%) had Lp(a) ≥60 and <100 mg/dl and 243 (10.5%) had Lp(a) ≥100 mg/dl.

**Table 1:** Main characteristics of the study population.

Parameter		Total subjects	Subjects with Lp(a) <60 mg/dl	Subjects with Lp(a) ≥60 mg/dl	P value (Lp(a) <60 vs. ≥60 mg/dl)
Sample size		2321	1811 (78.0%)	510 (22.0%)	
Male		1248 (53.8%)	977 (53.9%)	271 (53.1%)	0.75*
Age (years)		56.9±12.4	56.9±12.3	57.0±12.6	0.96**
Smoking	Never	1634 (70.4%)	1269 (70.1%)	365 (71.6%)	
	Former	198 (8.5%)	156 (8.6%)	42 (8.2%)	
	Current	488 (21.0%)	385 (21.3%)	103 (20.2%)	
Body mass index (kg/m^2^)		27.0±4.4	27.2±4.5	26.4±4.1	0.0003**
Atherosclerotic disease		473 (20.4%)	331 (18.3%)	142 (27.8%)	<0.0001*
Total cholesterol (mg/dl)		257±63	255±63	262±61	0.022**
LDL cholesterol (mg/dl)		162±50	160±50	168±49	0.003**
HDL cholesterol (mg/dl)		59±17	56±17	59±17	0.0003**
Triglycerides (mg/dl)		204±226	210±236	184±184	0.0004**
	Median (IQR)	149 (129)	153 (131)	135 (117)	
Lipoprotein(a) (mg/dl)		38±51	17±15	113±61	<0.0001**
	Median (IQR)	19 (46)	13 (19)	96 (63)	
Lipid lowering medication		229 (9.9%)	181 (10.0%)	48 (9.4%)	0.19*
Systolic blood pressure (mmHg)		137±19	137±19	136±20	0.74**
Diastolic blood pressure (mmHg)		83±10	83±10	82±10	0.60**
Hypertension		1139 (49.0%)	888 (49.0%)	251 (49.2%)	0.94*
Diabetes mellitus		394 (17.0%)	329 (18.2%)	65 (12.7%)	0.004*

Quantitative variables are presented as mean ± standard deviation and median and interquartile range (IQR) where appropriate; counts are given as *n* and percent.

*Chi-square P value.

**Unpaired Student's *t*-test (the data of Lp(a) and triglycerides were log-transformed before analysis).

### Genotyping of the −174G/C polymorphism in the study cohort

The allele frequencies of the G and C allele in the whole study population were 59.1% G and 40.9% C, respectively, whereas the matching genotype frequencies were 33.7% GG, 50.75% GC and 15.55% CC. The data are shown in **Table 2**. Genotype frequencies in the total study population were marginally departing from Hardy-Weinberg equilibrium (chi square P = 0.017), while the two groups considered separately were in agreement with HWE (low Lp(a) group chi square P = 0.08, high Lp(a) group chi square P = 0.06).

**Table 2:** Genotype distribution of the −174G/C SNP between subjects with lower (<60 mg/dl) or higher (≥60 mg/dl) Lp(a) levels.

Subjects	GG	GC	CC	Chi square P value
Total cohort (*n* = 2321)	782 (33.7%)	1178 (50.75%)	361 (15.55%)	0.024
Lp(a) <60 mg/dl (*n* = 1811)	635 (35.1%)	905 (50.0%)	271 (14.9%)	
Lp(a) ≥60 mg/dl (*n* = 510)	147 (28.8)	273 (53.5%)	90 (17.6%)	

	GG	C-carriers	
Total cohort (*n* = 2321)	782 (33.7%)	1539 (66.3%)	0.008
Lp(a) <60 mg/dl (*n* = 1811)	635 (35.1%)	1176 (64.9%)	
Lp(a) ≥60 mg/dl (*n* = 510)	147 (28.8%)	363 (71.2%)	

### Influence of the −174G/C SNP on Lp(a) concentrations

In the total cohort, there were differences in Lp(a) concentrations depending on genotype. The mean ± SD concentrations were 35.2±46.9 mg/dl, 37.9±51.2 mg/dl, and 40.3±55.3 mg/dl for the GG, GC, and CC genotype, respectively. The corresponding geometric means (95% CI) were 15.9 (14.4 to 17.5) mg/dl, 16.9 (15.6 to 18.3) mg/dl, and 18.5 (15.9 to 21.4) mg/dl, respectively (P for trend 0.042). We analyzed the effects of the −174G/C SNP on Lp(a) concentration using logistic regression analysis. The GC and CC genotypes were associated with a higher odds ratio for the prevalence of elevated Lp(a), defined as concentrations ≥60 mg/dl. For GC the odds ratio was 1.30 (95% CI 1.04 to 1.63, P = 0.02) and for CC the odds ratio was 1.44 (95% CI 1.06 to 1.93, P = 0.018). When taken together, the C-carriers had an odds ratio of 1.33 (95% CI 1.08 to 1.65, P = 0.009) compared to the GG wildtype.

### Influence of the −174G/C SNP on other parameters

Using analysis of variance we investigated whether the polymorphism influenced other continuous parameters. For simplicity, we divided the subjects in the ones having the GG wildtype (reference group) and the ones carrying the C-allele. The polymorphism was not associated with age (P = 0.98), BMI (P = 0.14), total cholesterol (P = 0.92), LDL cholesterol (P = 0.63), HDL cholesterol (P = 0.44), systolic or diastolic blood pressure (P = 0.19 or 0.36, respectively). It was significantly associated with triglycerides (P = 0.04), in that C-carriers had slightly higher triglyceride levels.

Using contingency tables and chi square statistics we investigated the association between the polymorphism and categorical data. There was no association with the presence of atherosclerosis (P = 0.62), diabetes (P = 0.61), or hypertension (P = 0.17).

### Multiple logistic regression analysis

In multiple logistic regression analysis using the presence of high Lp(a) concentrations as the dependent and age, sex, smoking, BMI, lipid concentrations, hypertension and diabetes as covariates we found that the polymorphism remained independently associated with high Lp(a) concentrations (odds ratio of the C-carriers 1.35, 95% CI 1.08 to 1.69, P = 0.009). The only significant covariate was LDL that also exerted an independent significant influence on Lp(a) concentrations.

## Discussion

The main finding of this study is that the common −174G/C polymorphism of the IL-6 gene is significantly associated with the risk of elevated Lp(a) concentrations (≥60 mg/dl).

Low grade inflammation has been shown to be important in the pathogenesis of atherosclerosis in cell lines, animal models and humans [20]. Of the many cytokines examined in this context, IL-6 exhibits a strong association not only with cardiovascular mortality itself [21] but also with cardiovascular risk factors like type 2 diabetes [22], [23], hypertension [24] and lipid abnormalities [25].

IL-6 is a pro-inflammatory cytokine with a molecular weight of 26 kD. The receptor of this signaling protein consists of two subunits, gp130 and IL-6 receptor-alpha (IL-6-R-α), and is linked to the *Janus kinase* (JAK) – *signal transducer and activator of transcription* (STAT) signaling cascade [26]. Interestingly, in addition to this classical signaling pathway, IL-6 can also act via the so called “trans-signaling”, whereby IL-6 binds to a soluble IL-6-R-α, thus activating cells only expressing the gp130 protein at the cell surface [27]. Furthermore, it has been shown that the *suppressor of cytokine signaling* (SOCS)-3, a protein known to impair insulin signaling, is induced by the JAK-STAT signaling pathway, suggesting a molecular mechanism by which IL-6 exerts metabolic effects [28].

A common SNP in the human IL-6 gene, −174G/C, has been associated with altered serum levels of this pro-inflammatory cytokine in humans, with carriers of the C-allele exhibiting higher serum levels of IL-6 in some [29] but not all [30] studies. However, whether the −174G/C polymorphism is associated with IL-6 concentrations is unclear. A number of studies examining this association produced widely discrepant results with some studies showing an association [29], [31]–[34], while many did not [30], [35]–[37]. Also, carriers of the C-allele show significantly higher C-reactive protein (CRP) levels compared to carriers of the G-allele [38], which is of interest since IL-6 is one of the major determining factors of CRP levels in humans. In agreement with the association studies on IL-6 serum levels and cardiovascular disease mentioned above, carriers of the C-allele have been shown to have a higher risk for coronary heart disease [39] and stroke [40] and also show a higher prevalence of risk factors like hypertension [41] and type 2 diabetes [42]. Moreover, in a cohort of 285 nonagenarians, the frequency of the “protective” G-allele was significantly associated with longevity, indicating a clinical significance of this common IL-6 polymorphism [43].

Using the humanized IL-6 receptor antibody tocilizumab we recently demonstrated an approximately 30% reduction of serum Lp(a) levels due to inhibition of IL-6 signaling in humans [8]. The higher frequency of the C-allele at the −174 locus in the IL-6 gene in human subjects with elevated Lp(a) levels reported here is in complete agreement with these findings, since the C-allele at this locus is associated with higher IL-6 serum levels [29]. Furthermore, an earlier study suggested regulation of Lp(a) serum levels by *acute phase response* (APR), for which IL-6 is crucial [44]. Taken together, these findings suggest that IL-6 is a major determining factor of serum Lp(a) levels in human subjects.

Despite evidence from previous reports [42], we did not find in our cohort a significant association of the −174G/C polymorphism with type 2 diabetes. In this respect it is important to note that our cohort was not collected to examine genetic influence on diabetes development but to identify target genes involved in severe dyslipidemia. Therefore, the study cohort does not reflect a representative population of type 2 diabetic subjects. Furthermore, it should be mentioned that an independent association of the −174G/C polymorphism with type 2 diabetes risk was found in several reports, but not in the largest of such studies performed in Denmark in 2005 [9]. Therefore, the potential role of the −174G/C SNP in the pathogenesis of type 2 diabetes remains controversial. Moreover, we found no association of the SNP with BMI, which is in agreement with previous reports [45].

Besides influencing Lp(a) levels, we also found significantly higher serum triglyceride concentrations in carriers of the C-allele at the −174 locus of the human IL-6 gene. This is of interest, since studies in rodents suggest that IL-6 treatment increased hepatic triglyceride secretion without decreasing the clearance of triglyceride-rich lipoproteins [46]. Furthermore, apolipoprotein B, which is important for the formation of very-low-density lipoprotein (VLDL) particles, has been found to be secreted from hepatocytes in response to IL-6 treatment [47]. Moreover, our findings are in agreement to those of Henningsson et al., who showed that male carriers of the C allele displayed elevated serum triglycerides [48]. We also observed that Lp(a) levels are inversely associated with the risk of type 2 diabetes, an interesting finding recently also reported by the group of Ridker et al. [49]. Moreover, we observed that Lp(a) concentrations are associated with LDL-, total- and HDL-cholesterol concentrations, in accordance to previous reports [50], [51]. The inverse association between triglyceride levels and BMI with Lp(a) levels found in the present study is in agreement with the findings of Chien et al. [52], although the association of Lp(a) levels with BMI remains controversial [53].

Taken together, the positive association of the C-allele at the −174 locus of the human IL-6 gene with elevated Lp(a) concentrations together with the Lp(a)-decreasing effects of the IL-6 antagonist tocilizumab strongly suggests that Lp(a) serum levels are regulated by IL-6 in humans. Since presently the number of drugs to treat elevated Lp(a) levels in patients with cardiovascular diseases is limited [54], these findings may open an interesting field for biomedical research in order to develop novel pharmacological strategies for affected patients.
